# Focus on the Tumour Periphery in MRI Evaluation of Soft Tissue Sarcoma: Infiltrative Growth Signifies Poor Prognosis

**DOI:** 10.1155/SRCM/2006/21251

**Published:** 2006-12-06

**Authors:** Josefin Fernebro, Marie Wiklund, Kjell Jonsson, Pär-Ola Bendahl, Anders Rydholm, Mef Nilbert, Jacob Engellau

**Affiliations:** ^1^Department of Oncology, Institute of Clinical Sciences, Lund University Hospital, 221 85 Lund, Sweden; ^2^Department of Diagnostic Radiology, Institute of Clinical Sciences, Lund University Hospital, 221 85 Lund, Sweden; ^3^Department of Orthopedics, Institute of Clinical Sciences, Lund University Hospital, 221 85 Lund, Sweden

## Abstract

*Purpose*. Infiltrative microscopical peripheral growth of soft tissue sarcomas (STS) has been shown to be of prognostic importance and preoperative risk stratification could individualize neoadjuvant treatment. *Patients and methods*. We assessed peripheral tumour growth pattern on preoperative MRI from 78 STS. The findings were correlated to histopathology and to outcome. *Results*. The MRI-based peripheral tumour growth pattern was classified as pushing in 34 tumours, focally infiltrative in 25, and diffusely infiltrative in 19. All tumours with diffuse infiltration on MRI also showed microscopical infiltration, whereas MRI failed to identify infiltration in two-thirds of the microscopically infiltrative tumours. Diffusely infiltrative growth on MRI gave a 2.5 times increased risk of metastases (*P* = .01) and a 3.7 times higher risk of local recurrence (*P* = .02). *Discussion*. Based on this observation we suggest that MRI evaluation of STS should focus on the peripheral tumour growth pattern since it adds prognostic information of value for decisions on neoadjuvant therapies.

## INTRODUCTION

Soft tissue sarcomas (STS) are rare and heterogenous tumours that often require combination therapy. Despite multidisciplinary and multimodality treatment, 10–20% of the tumours recur locally and distant metastases develop in about 30% of the patients [1, 2]. Various prognostic systems are in use, most of which are based on combinations of tumour size, histologic malignancy grade, necrosis, and vascular invasion [1, 3, 4]. Tumour size can be determined by preoperative imaging, whereas preoperative assessment of the other factors will be based on the limited biopsy material. MRI is the imaging modality that is most frequently used not only for preoperative evaluation of tumour size but also for the mapping of the anatomical extension of STS [5–7]. Prognostic factors that can be evaluated preoperatively would be clinically valuable in order to identify high-risk patients for neoadjuvant radiotherapy and chemotherapy.

We have recently shown that STS with a microscopically infiltrative growth pattern, as determined on whole-tumour sections, have a considerably higher risk (HR 4.6) for both local recurrence and metastasis compared to STS with a pushing growth pattern [8]. The prognostic strength of infiltrative growth was similar to or stronger than that of other commonly used prognostic factors. The possibility to identify infiltrative growth of STS on MRI has, to our knowledge, not yet been assessed and we therefore aimed to evaluate the peripheral tumour growth pattern on preoperative MRI sequences and correlated these findings to the microscopical characteristics on whole-tumour sections and to outcome in 78 patients with STS of the extremities and the trunk wall.

## PATIENTS AND METHODS

### Patients

This retrospective investigation was based on adult (> 18 years) patients treated at the Musculoskeletal Tumour Centre in Lund between 1989 and 2000. Patients with primary STS of the extremities or the trunk wall who had been referred to our centre before any surgery and who had no detectable metastases at the time of diagnosis were eligible for the study. In addition, preoperative MRI scans should be available and neoadjuvant chemotherapy or radiotherapy should not have been administered. In order to compare the growth patterns on MRI and on microscopical evaluation, the tumours should have been resected with a marginal or a wide surgical margin and whole-tumour sections should be available. Hereby, we have identified 78 patients, which represent a subset of the 140 patients in whom we have previously reported the prognostic value of microscopical infiltrative growth on whole-tumour sections [8]. The main reason for exclusion from the former series was that only preoperative CT had been performed. The lower extremity was the most common tumour location, 2/3 of the tumours were deep-seated, leiomyosarcoma was the commonest histiotype and 66 of the 78 tumours were high-grade (grades 3 and 4 on a 4-tiered scale) (Table 1). Follow-up was complete for at least 5 years for the survivors. Local recurrences developed in 13/78 (rate 0.2) patients and metastases in 33/78 (rate 0.4) patients.

### Microscopic assessment based on whole-tumour sections

**Figure 1:**
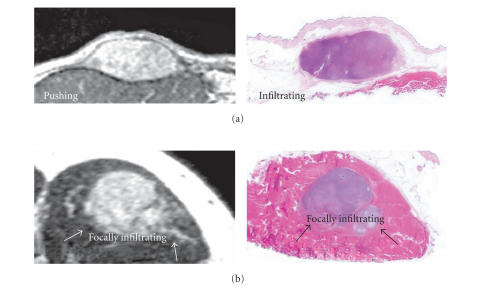
Examples of MRI scans and whole-tumour sections stained with haematoxylin and erythrosine from 2 different leiomyosarcomas; (a) a subcutaneous tumour of the thigh with a pushing growth pattern on MRI, but microscopic infiltration on histopathology, (b) an intramuscular tumour of the thigh with focally infiltrative growth pattern on both MRI and histopathology.

This evaluation is a continuation of a previously published study [8]. In short, a whole-tumour section was obtained from the maximum tumour diameter and was, after dehydration, embedded into paraffin. The microscopical assessment was performed on a 4 *μ*m slide stained with haematoxylin-erythrosin. The peripheral tumour growth pattern was microscopically classified as pushing in 22 tumours, where no sign of infiltrative growth could be detected, or else as infiltrative in 56 tumours (infiltration involved < 25% of the tumour rim in 14 tumours and > 25% in 42 tumours, without differences in outcome between these groups, which were subsequently combined) (see Table 2 and Figure 1). In this subset of 56 tumours, infiltrative growth predicted risk of metastases and local recurrence similarly to previously reported [8] with an HR of 3.7 (95% CI 1.3–11, *P* = .01) for development of metastases and with all local recurrences occurring among infiltrative tumours (*P* = .009; log-rank test).

### Assessment of preoperative MRI

All patients had undergone preoperative MRI, most of them at local hospitals before referral to our musculoskeletal tumour center. Different MRI equipment, including low-Tesla units as well as 1.5-Tesla units were used. Standard MRI included axial and coronal sections, and in some cases also sagittal sections, with T1- and T2-weighted sequences, coronal STIR sequence, and a static T1-weighted fat saturated sequence after intravenous contrast medium injection, most often gadolinium DTPA. The MRI examinations were retrospectively evaluated in consensus by two musculoskeletal radiologists (MW and KJ) who were blinded to the histopathological data and the outcome data. The assessment of peripheral growth pattern on MRI was based on the largest midsection of the tumour. Pushing growth pattern was considered when the tumour was well defined without peripheral extension to the surrounding tissue, whereas classified as infiltrative if the tumour had an irregular surface with spicula-like extensions into the surrounding tissue. Infiltrative growth was classified as focal (< 25% of the tumour circumference) or diffuse (> 25% of the circumference) (Figure 1).

### Statistical analysis

Associations between categorical or categorized variables were evaluated with chi-squared tests. Time to first metastasis and to first local recurrence was analyzed using Kaplan-Meier estimates, log-rank tests, and Cox regression. Proportional hazards assumptions were checked graphically. All tests were two-sided and the significance level was set to .05. We used the statistics package Stata 9.0 (StataCorp 2005, College Station, Tex).

## RESULTS

All 22 tumours with a pushing growth pattern on histopathology were classified as pushing or focally infiltrative on MRI. 4 of these tumours developed metastasis and none recurred locally. Among the 56 microscopically infiltrative tumours, MRI identified 37 as pushing or focally infiltrative, and 19 as diffusely infiltrative (Table 2). The MRI-based growth pattern showed no obvious association with tumour size, depth, or grade (Table 2); indeed a pushing growth pattern was identified in 2/3 of the large (> 5 cm) tumours and in 1/3 of the grade IV tumours.

**Figure 2:**
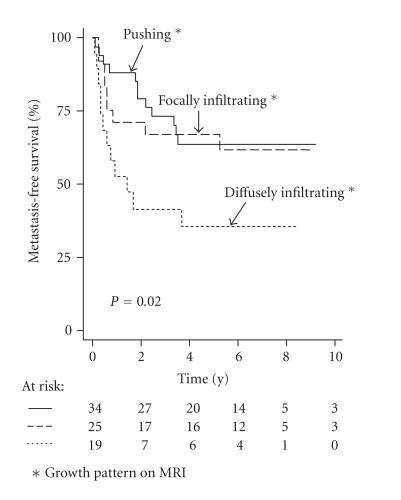
Kaplan-Meier survival curves in relation to infiltrative growth identified on MRI (*P* = .02).

Metastases developed in 12 of the 19 (rate 0.6) diffusely infiltrative tumours compared to 21/59 (rate 0.4) in the tumours with a pushing or focally infiltrative growth pattern on MRI (*P* = .03). The former group had an HR of 2.5 for the risk of development of metastases (95% CI = 1.2–5.1; *P* = .01) (Figure 2). Local tumour recurrences developed in 6/19 (rate 0.3) diffusely infiltrative tumours compared to 7/59 (rate 0.1) tumours with a pushing or focally infiltrative growth pattern on MRI (*P* = .04). In the analysis of local recurrence free survival, infiltrative growth on MRI showed an HR of 3.7 (95% CI = 1.2–11; *P* = .02).

## DISCUSSION

MRI have become part of the standard procedure for the preoperative evaluation of STS because of high-resolution mapping of the anatomical extension of the tumour. Dynamic, contrast-enhanced MRI has been suggested to differentiate viable from nonviable (necrotic or avascular) tumour areas, and could therefore potentially be valuable for preoperative prognostication [9]. The overall, prognostic value of preoperative MRI, however, is largely unknown. We have in a recent study demonstrated that the microscopical peripheral tumour growth pattern (pushing versus infiltrative) determined on whole-tumour sections provides new and independent prognostic information in STS; tumours with infiltrative growth have an increased risk for local recurrences as well as for metastases [8]. We did not analyse the relation between MRI findings and prognostic factors such as tumour size and histologic malignancy grade since our aim was to assess whether growth pattern on MRI could be used for prognosis rather than to assess a value of combining MRI findings with other factors in a prognostic system for soft tissue sarcoma.

In the current study we found that tumours with diffuse infiltrative growth on MRI had a worse prognosis, both with regards to local recurrence and metastasis, whereas tumours with a pushing or focally infiltrative growth pattern on MRI had a better prognosis. In the latter groups MRI was less accurate; almost one third of the histopathologically pushing tumours had focal infiltration on MRI and one third of the histopathologically infiltrative tumours had pushing growth on MRI. In our previous study [8] (see Material and Methods) we found that the percentage of the tumour rim that was histopathologically infiltrative was of no prognostic importance; tumours were divided into pushing tumours, with no infiltration anywhere, versus all others. In the current study we found the inverse; only diffuse infiltration on MRI was of prognostic importance. Difficulties in determining infiltration on MRI probably account for this difference; the diffusely infiltrating tumours (with infiltration around the entire tumour rim) are more likely to identify infiltrative growth as determined histopathologically, whereas focal infiltration on MRI may be false positive.

We recognize several weaknesses in our study, namely, retrospective analysis and nonstandardized MRI examinations. The resolution was suboptimal in several cases, which may reflect nonstandardization as well as MRI having a lower resolution than microscopic examination and this may explain why infiltration was not identified in many tumours and classified as focally infiltrative in many tumours with a pushing growth on histopathology. However, our finding of a poor prognosis for tumours with diffuse infiltration on MRI suggests that MRI should in a standardized way classify the peripheral tumour growth pattern. We therefore suggest that our findings should be tested in a prospective study using high-resolution MRI in order to improve the preoperative risk assessment in patients with STS.

## Figures and Tables

**Table 1:** Clinical pathological characteristics in 78 soft tissue sarcomas.

Age	
Median (range) years	68 (23–87)

Site	
Upper extremity	15
Lower extremity	59
Trunk wall	4

Size	
≤ 5 cm	23
> 5 cm	55

Microscopical diagnosis	
Leiomyosarcoma	25
Pleomorphic/unclassified STS	12
Liposarcoma	12
Myxofibrosarcoma	10
MFH*	9
#Other	10

Histological malignancy grade (numbers within parenthesis refer to patients who developed metastases)	
I/II	12 (2)
III	15 (7)
IV	51 (24)

Tumour depth	
Subcutaneous	23
Deep-seated	55

Local treatment (numbers within parenthesis refer to patients who developed local recurrences)	
Marginal	9 (2)
Marginal with radiotherapy	24 (5)
Wide**	45 (6)

*MFH, malignant fibrous histiocytoma.

#Includes neurofibrosarcoma, MPNST, synovial sarcoma, extraskeletal chondrosarcoma, and angiosarcoma.

**Radiotherapy administered to two patients.

**Table 2:** Correlations between MR findings and clinicopathological data.

MR classification	Pushing		Focally infiltrative		Diffusely infiltrative	

Histopathologic growth pattern						
Pushing	14	2 met*, 0 lr**	8	2 met, 0 lr	0	—
Infiltrating	20	10 met, 3 lr	17	7 met, 4 lr	19	12 met, 6 lr

Size						
≤ 5 cm	11	—	8	—	4	—
> 5 cm	23	—	17	—	15	—

Depth						
Subcutaneous	11	—	5	—	7	—
Deep-seated	23	—	20	—	12	—

Grade						
I/II	9	—	3	—	0	—
III	6	—	3	—	6	—
IV	19	—	19	—	13	—

*met = metastasis.

**lr = local recurrence.
